# Patterns of Nicotine Use Among Women in the Rural Population of Kolar: A Cross-Sectional Study

**DOI:** 10.7759/cureus.65698

**Published:** 2024-07-29

**Authors:** Stuti J Mashru, MohanReddy Matti, Ruth Sneha

**Affiliations:** 1 Psychiatry, Sri Devaraj Urs Academy of Higher Education and Research, Kolar, IND

**Keywords:** smokeless tobacco(st), from india, fagerstrom test for nicotine dependence, rural women, smokeless tobacco

## Abstract

Background

The prevalence of smokeless tobacco (SLT) use among Indian women, particularly those from lower socioeconomic backgrounds with little access to formal education, has been steadily increasing, which is a cause for concern. Women frequently use various forms and companies of powdered, rubbed, and chewed SLT, with or without betelnut and flavorings, often simultaneously, starting at an early age and persisting into their reproductive years. Consequently, they are vulnerable to both the chance of developing cancer and experiencing health issues during pregnancy and childbirth. The purpose of the study was to assess the prevalence of women using SLT and the factors that were linked to these outcomes.

Methodology

The community-based analytical, cross-sectional study was carried out for four months (February-May 2024) in the selected rural areas of Kolar district, Karnataka, India. Women aged 15 years and above who lived in the selected rural areas of Kolar district as permanent residents and had a history of nicotine use were included. Women using smoked forms of tobacco and bedridden or terminally ill cancer patients were excluded from the study. An interviewer administered a semistructured interview schedule to collect data. The questionnaire included sections on sociodemographic characteristics (age, education, occupation, marital status, socioeconomic status, and type of family), nicotine use patterns (types of SLT/nicotine products used and mode of use), maternal history, menstrual history, alcohol consumption history, morbidity conditions, and nicotine dependence, which were assessed by Fagerstrom Test for Nicotine Dependence (FTND).

Results

The participants, 92 women, ranged in age from 15 to 80 years old, with a mean age of 41.2 years. Most of the participants were diagnosed with oral cancer (n = 19, 20.7%), followed by esophageal cancer (n = 13, 14.1%). When participants were enquired about the mode of usage of tobacco, most of them used chewable form (n = 43, 46.7%). When dependence was assessed by the Fagerstrom nicotine dependence scale, high dependence was observed in 83.7% of women (n = 77), whereas low-to-moderate dependence was observed in 16.3% of women (n = 15). Among the 92 participants, only 16 participants (17.3%) made attempts to quit using SLT. The sociodemographic factors associated with nicotine dependence included age between 41 and 60 years, illiteracy, lower economic status, widowhood, unmarried status, unemployment, Hindu by religion, nuclear family, non-alcoholic, irregular menstrual cycles, and significant maternal history (P-value less than 0.05).

Conclusion

The development of tailored interventions that address the specific needs of illiterate, unemployed, lower-class, and unmarried women in nuclear families was observed. These interventions should incorporate mental health screening, psychoeducation, and community-based support to promote cessation of SLT and improve their overall well-being.

## Introduction

An ongoing global issue we encounter is the prevalence of smokeless tobacco (SLT) consumption [1]. The use of tobacco by women is increasingly receiving increased attention. Data on tobacco usage among women from low- and middle-income nations are limited. These nations have low rates of female smoking prevalence, which may result from under-reporting [2]. Males use SLT at higher rates than females among adults; nevertheless, in certain nations, like Bangladesh, Thailand, Cambodia, Malaysia, Vietnam, South Africa, Mauritania, Sierra Leone, and Barbados, usage of SLT is equal to or higher in females than in males. There are a variety of cultural, psychological, and socioeconomic factors that may contribute to tobacco usage. The customs surrounding tobacco smoking vary from area to region in India because of differences in family life, social standards, and cultural influences on behavior [3].

The prevalence of SLT consumption among Indian women, particularly those from lower socioeconomic backgrounds and with limited access to formal schooling, has been steadily increasing, causing concern. Women frequently use various forms and brands of powdered, rubbed, and chewed SLT, with or without betelnut and flavorings, often simultaneously, starting at an early age and persisting into their reproductive years [4]. There are worries that women in rural regions are more likely to use tobacco than males, as evidenced by a study of tobacco usage in seven states and union territories, including Karnataka. This suggests that youngsters are being conditioned to use nicotine at an earlier age. The researchers observe that, according to the most recent NFHS-5 (National Family Health Survey) data (2019-2020), tobacco usage among women in rural India has increased from 13.67% in NFHS-4 to 17.83% in NFHS-5 in a study published by the Delhi-based think tank AF Development Center (AFDC).

Although the data shows that the increase is seen in all states except for Manipur, Meghalaya, and Nagaland, the report concentrates on Karnataka, Assam, Gujarat, Odisha, Uttar Pradesh, and Jammu and Kashmir. The director of AFDC and senior investigator, Sachi Satapathy, stated that the greatest rates of cigarette smoking were found in Odisha and Karnataka, at 62.50% and 63.68%, respectively [5].

As it contains carcinogens and exposes users to the danger of leucoplakia, which includes precancerous lesions and oral cancer, SLT can cause gum and mouth diseases. SLT usage during pregnancy increases the risk of stillbirth and premature delivery in women [6]. Women are more vulnerable to the negative physical, psychological, and social effects of substance use disorders than men. This is besides having a shorter latent time between the start of substance use and the development or progression of the diseases, according to epidemiological analysis of many studies [7,8]. Children are also affected by this inadvertently. According to certain reports, compared to 5% of men, 18% of women send their kids to buy tobacco goods. Researchers warned that this causes children get exposed to nicotine products at a young age. Since the shop owners are prohibited from selling tobacco goods to customers under the age of 18, this further shows that they are not abiding by the law [5].

There is a notable dearth of information addressing epidemiological data and hazards as well as understanding among women concerning tobacco [9]. Providing sufficient evidence on the community's correlations with tobacco use has become imperative to support policy makers, healthcare providers, and the public in creating workable models for tobacco control that will serve the needs of various underserved communities. The cross-sectional design was conducted to assess the pattern of nicotine use among rural women in Kolar district in Karnataka and to determine the association between sociodemographic profile and SLT dependence.

## Materials and methods

Study design, period, and participants

The community-based analytical, cross-sectional study was carried out for four months (February-May 2024) in the selected rural areas of Kolar district, Karnataka, India. Narsapura, Vemagal, Sugatur, Vokkaleri, and Tekal are the rural areas in Kolar district that we selected. These rural areas were the rural field practice area of RL Jalappa Hospital, affiliated with Sri Devaraj Urs Medical College in Tamaka, Kolar, India. The target population included women who had used SLT in any form.

Inclusion criteria

Women aged 15 years and above who were living in the selected rural areas of Kolar district as permanent residents and had a history of nicotine use in women were included. Patients who were willing to participate or provide written informed consent for the study were included.

Exclusion criteria

Women using smoked forms of tobacco and bedridden or terminally ill cancer patients were excluded from the study.

Sample size and sampling method

The sample size was calculated based on an estimated prevalence of nicotine use among rural women, with a desired level of precision and confidence interval. As per the study conducted by Dasgupta et al., which states that the prevalence of SLT consumption is 36.2% [10], sample size calculation was done using the formula: N = 3.84 x p x q/d^2^, where p denotes prevalence, q denotes the complement of p, and d denotes precision (with a 10% absolute error). We calculated the sample size according to the above prevalence rate and 95% confidence interval. It was determined that the study required at least 89 samples. A convenient sampling technique was used to select the required samples from the study area.

Data collection procedure

A semistructured interview schedule was administered by an interviewer to collect data. This face-to-face interview was conducted by trained interviewers fluent in the local language (Kannada). The questionnaire included sections on sociodemographic characteristics (age, education, occupation, marital status, socioeconomic status, and type of family), nicotine use patterns (types of SLT/nicotine products used and mode of use), maternal history, menstrual history, alcohol consumption history, morbidity conditions, and nicotine dependence, which were assessed by Fagerstrom Test for Nicotine Dependence (FTND).

Operational definitions

Smokeless Tobacco

SLT can be used orally or nasally and is consumed without burning the product. Oral SLT products are chewed or sucked into the mouth, cheek, or lip. Applying tobacco pastes or powders to the gums or teeth works similarly. Typically, inhaled fine tobacco mixtures are absorbed through the nasal passages [11].

Fagerstrom Test for Nicotine Dependence (FTND)

A common tool for determining the degree of physical addiction to nicotine is the FTND. The Yes/No questions on the FTND are scored between 0 and 1, and the multiple-choice questions are scored between 0 and 3. The items' sums result in a final score ranging from 0 to 10. The degree of the patient's physical dependence on nicotine increases with the total Fagerstrom score [12].

Ethical approval

Before data collection, ethical approval was obtained from the Institutional Review Board of RL Jalappa Hospital & Research centre, Tamaka, Kolar (approval number: SDUAHER/KLR/R&D/CEC/S/PG/16/2024-25). Informed consent was obtained from each participant before the interview. Participants were assured of confidentiality, and their participation was voluntary.

Statistical analysis

All data were entered in Excel (Microsoft® Corp., Redmond, WA) and analyzed in IBM SPSS Statistics for Windows, version 26.0 (released 2019, IBM Corp., Armonk, NY). Frequency and percentage were employed to summarize categorical variables such as religion, educational status, marital status, etc. Using the chi-square test, the association between the various sociodemographic factors and SLT dependence was assessed. A p-value was considered significant if it was less than 0.05.

## Results

The participants, 92 women, ranged in age from 15 to 80 years, with a mean age of 41.2 years. About 90.2% of the population identified as Hindu. Respondents' educational levels were evenly distributed, with the majority (23.9%) being uneducated and the same percentage (23.9%) having graduated. Regarding employment status, 83.7% of the participants were unemployed. The sociodemographic characteristics of the participants are given in Table 1.

**Table 1:** Sociodemographic characteristics of the participants (n = 92)

Characteristics	Frequency	Percentage
Age group		
Less than 18 years	5	5.4%
18-30 years	7	7.7%
31-40 years	13	14.1%
41-50 years	47	52%
51-60 years	11	11.9%
More than 60 years	9	9.8%
Religion		
Hindu	82	89.1%
Muslim	10	10.9%
Educational status		
Uneducated	52	56.5%
Primary school	18	19.6%
High school	14	15.3%
Higher secondary	4	4.3%
Graduate	4	4.3%
Employment status		
Employed	9	9.7%
Unemployed	83	90.3%
Marital status		
Unmarried	38	41.3%
Married	21	22.8%
Widow	33	35.9%
Socioeconomic status		
Lower class	82	89.1%
Middle class	10	10.9%
Type of family		
Joint family	44	47.8%
Nuclear family	48	52.2%

**Figure 1:**
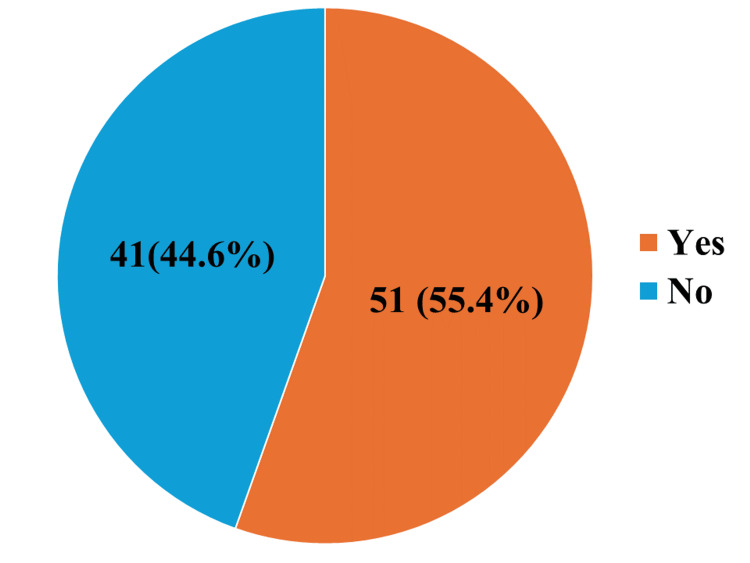
History of alcohol consumption among the participants (n = 92)

Figure 1 shows the history of alcohol consumption, which was recorded in 22.9% of the study participants.

**Figure 2:**
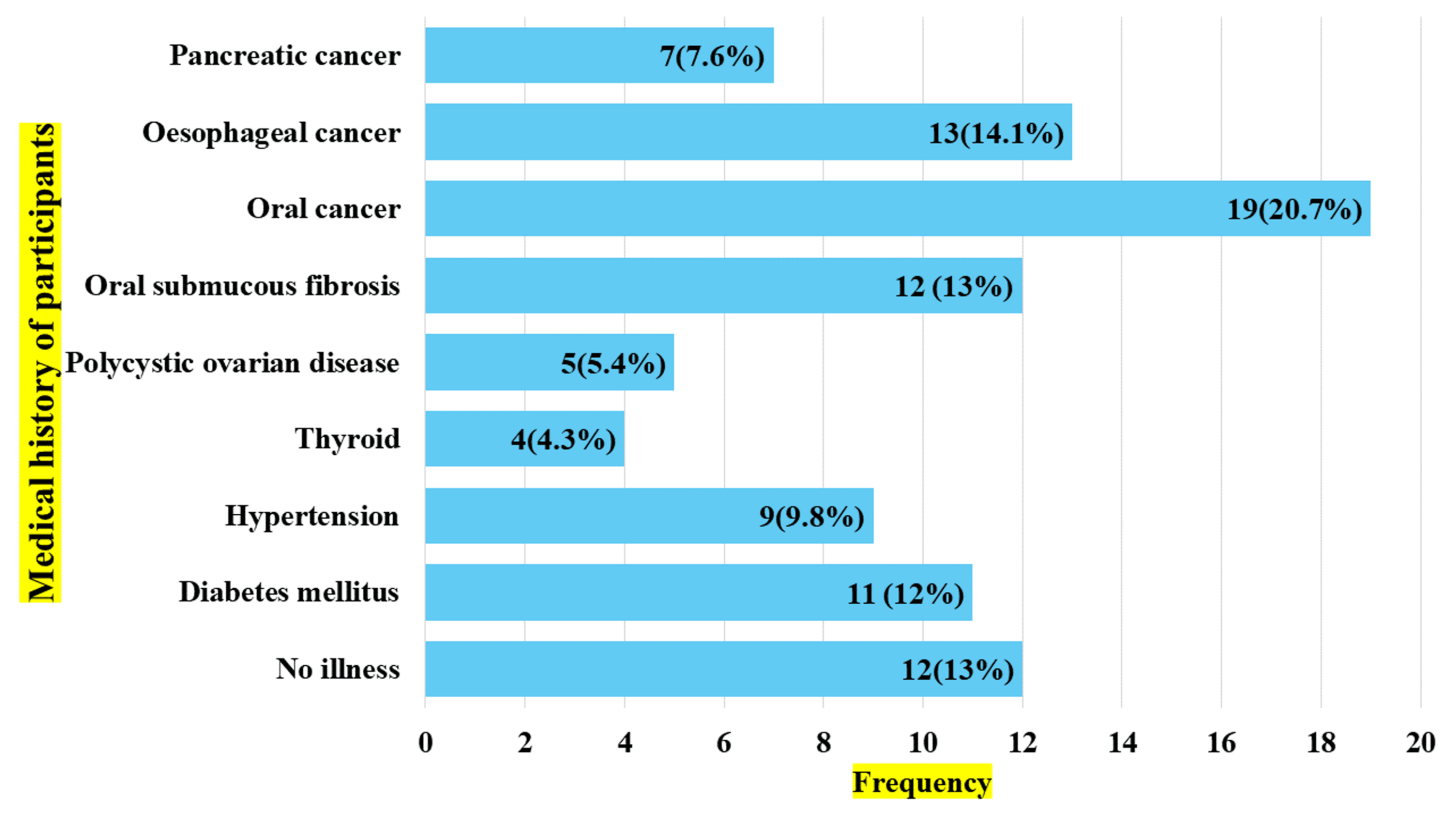
Medical history of the participants (n = 92)

When the medical history of the participants was questioned, most of the participants were diagnosed with oral cancer (n = 19, 20.7%), followed by esophageal cancer (n = 13, 14.1%). Approximately 13% of the participants (n = 12) had no history of illness. The distribution of the medical history of the participants is given in Figure 2.

**Figure 3:**
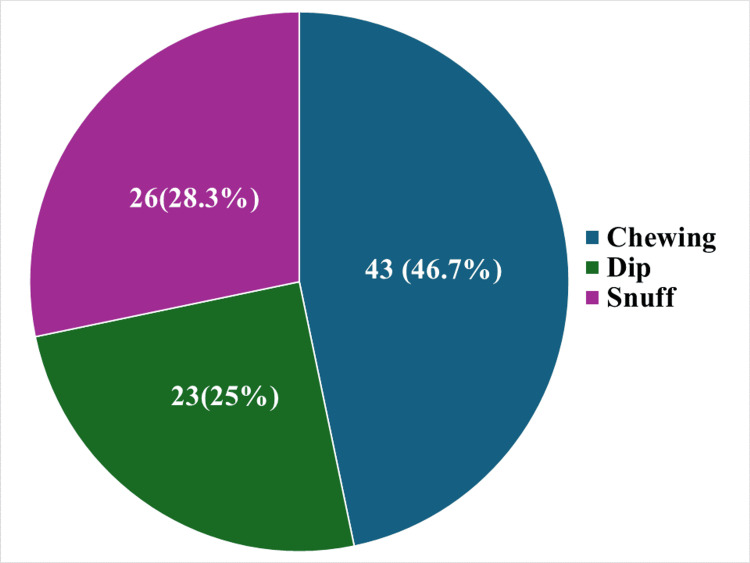
Mode of smokeless tobacco use (n = 92)

When participants were enquired about the mode of usage of tobacco, the majority of them used chewable form (n = 43, 46.7%). The mode of tobacco usage is shown in Figure 3.

**Figure 4:**
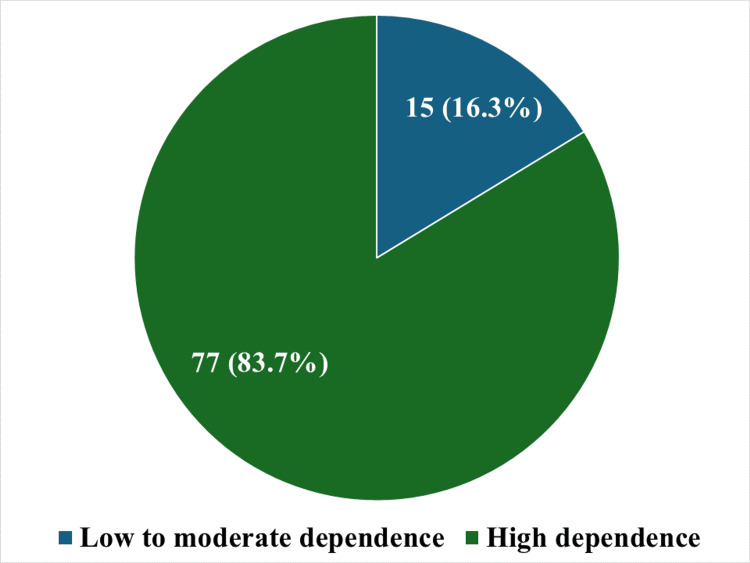
Prevalence of smokeless tobacco dependence among the participants based on Fagerstrom Nicotine Dependence Scale-smokeless tobacco (n = 92)

When dependence was assessed by the Fagerstrom nicotine dependence scale, high dependence was seen in 83.7% of women (n = 77), whereas low-to-moderate dependence was seen in 16.3% of women (n = 15). This has been represented in Figure 4.

Table 2 shows the menstrual and maternal history of study participants. When enquired about the menstrual history, 22.8% of the participants had regular cycles, 40.2% of the participants had irregular cycles, and 37% of the participants attained menopause. When maternal history was probed, 46.7% of the participants had no significant history, whereas preterm delivery, history of abortions, and infertility were present in 19.5%, 18.5%, and 15.2% of the participants, respectively.

**Table 2:** Menstrual and maternal history of study participants

Gynecological history	Frequency (n = 92)	Percentage (%)
Menstrual history		
Regular cycles	21	22.8
Irregular cycles	37	40.2
Postmenopausal women	34	37.0
Maternal history		
No significant history	43	46.7
Preterm delivery	18	19.6
Abortions	17	18.5
Infertility	14	15.2

**Figure 5:**
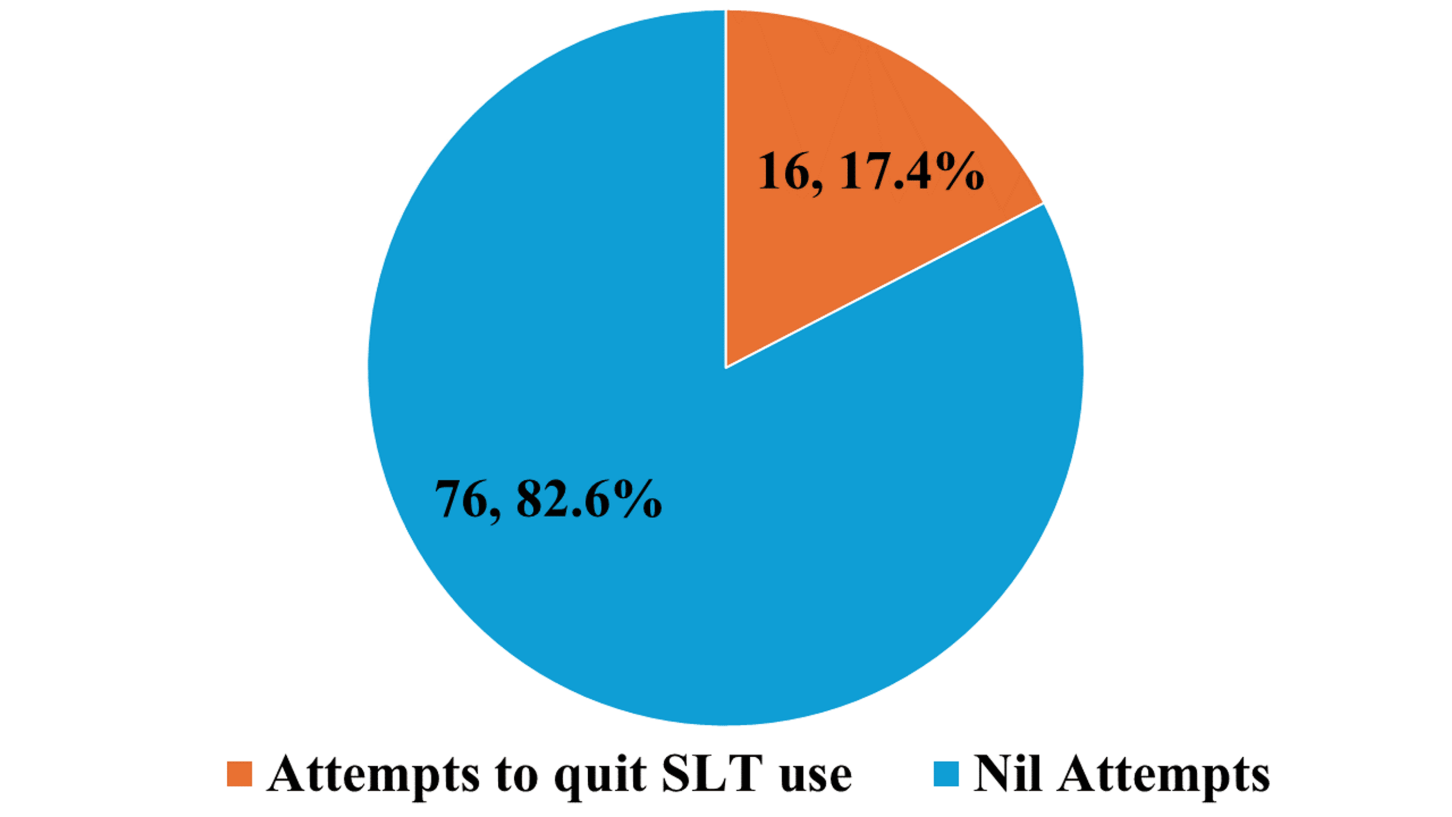
Percentage of the study participants' attempts to quit using smokeless tobacco

Figure 5 shows the percentage of study participants who attempted to quit using SLT. Among the 92 participants, only 16 participants (17.4%) made attempts to quit SLT. The reasons quoted for attempting to quit were financial reasons, health issues, and pressure from family members. But all these 16 participants had a relapse of the habit, for which stress and cravings were cited as a reason.

Table 3 shows the association between sociodemographic factors, maternal and menstrual history, and SLT dependence scores. Women less than 18 years and middle-aged women had high dependence, and the difference in proportion was found to be statistically significant by Fischer's exact test (p-value = 0.04). Similarly, uneducated women and women with primary education were also found to have high dependence, and the difference between the groups was also statistically significant (p-value = 0.01). Women belonging to the lower class, unmarried women, and uneducated women have also been found to have higher dependence, and the difference between the groups was also found to be statistically significant. Women of Hindu religion (p-value = 0.036) and women living in joint families (p-value < 0.01) were found to have higher levels of dependence, and the difference between the groups was statistically significant.

**Table 3:** Association of sociodemographic variables with levels of low-to-moderate and high dependence *Chi-square test was used. **Fischer's exact test was used.

Characteristics	Levels of dependence		Test value	P-value
	Low-to-moderate dependence (n = 15)	High dependence (n = 77)		
Age of the participants				
Less than 18 years	1 (20%)	4 (80%)	11.382	0.04**
18-30 years	3 (42.8%)	4 (57.2%)		
31-40 years	4 (30.7%)	9 (69.3%)		
41-50 years	3 (6.3%)	44 (93.7%)		
51-60 years	1 (9%)	10 (91%)		
More than 60 years	3 (33.3%)	6 (66.7%)		
Educational status				
Uneducated	3 (5.7%)	49 (94.3%)	12.368	0.01**
Primary school	4 (22.2%)	14 (77.8%)		
High school	6 (42.8%)	8 (57.2%)		
Higher secondary	1 (25%)	3 (75%)		
Graduate	1 (25%)	3 (75%)		
Socioeconomic status				
Lower class	11 (13.4%)	71 (86.6%)	4.616	0.03*
Middle class	4 (40%)	6 (60%)		
Marital status				
Unmarried	4 (10.5%)	34 (89.5%)	9.495	0.01*
Married	8 (38%)	13 (62%)		
Widow	3 (9%)	30 (91%)		
Employment status				
Employed	4 (44.4%)	5 (55.6%)	5.789	0.016*
Unemployed	11 (13.2%)	72 (86.8%)		
Religion				
Hindu	11 (13.3%)	72 (86.7%)	5.789	0.036*
Muslim	4 (44.4%)	5 (55.6%)		
Type of family				
Joint family	13 (29.5%)	31 (70.5%)	10.835	0.01*
Nuclear family	2 (4.1%)	46 (95.9%)		

Table 4 shows the association between alcohol consumption and SLT dependence scores. There was a statistically significant difference in the level of dependence observed among women who consume alcohol (p-value = 0.03).

**Table 4:** Association of alcohol consumption with levels of low-to-moderate and high dependence *Chi-square test was used.

Alcohol consumption	Levels of dependence		Test value	P-value
	Low-to-moderate dependence (n = 15)	High dependence (n = 77)		
Yes	12 (23.5%)	39 (76.5%)	4.378	0.03*
No	3 (7.3%)	38 (92.7%)		

Table 5 shows the association between medical illness and SLT dependence scores. There was no statistically significant difference in the level of dependence observed among women with medical illness (p-value = 0.668).

**Table 5:** Association of medical illness with levels of low-to-moderate and high dependence **Fischer's exact test was used. PCOD: Polycystic ovarian disease.

Medical illness	Levels of dependence		Test value	P-value
	Low-to-moderate dependence (n = 15)	High dependence (n = 77)		
Diabetes mellitus	3 (27.3%)	8 (72.7%)	5.744	0.668**
Esophageal cancer	4 (30.8%)	9 (69.2%)		
Hypertension	2 (22.2%)	7 (77.8%)		
Oral cancer	3 (15.8%)	16 (84.2%)		
Oral submucous fibrosis	1 (8.3%)	11 (91.7%)		
Pancreatic cancer	0 (0%)	7 (100%)		
PCOD	1 (20%)	4 (80%)		
Thyroid	0 (0%)	4 (100%)		
No illness	1 (8.3%)	11 (91.7%)		

Table 6 shows the association between medical illness and SLT dependence scores. Statistically significant results were observed among women with regular menstrual cycles and those in the postmenopausal age range, indicating a high level of dependency. The difference between these two groups was determined to be statistically significant, with a p-value of 0.04. Furthermore, it was observed that women with a high level of dependence had a greater incidence of premature birth, abortions, and infertility. The differences between the groups were also determined to be statistically significant, with a p-value of 0.04.

**Table 6:** Association of menstrual and maternal history with levels of low-to-moderate and high dependence *Chi-square test was used. **Fischer’s exact test was used.

Gynecological history	Levels of dependence		Test value	P-value
	Low-to-moderate dependence (n = 15)	High dependence (n = 77)		
Menstrual history				
Regular cycles	7 (33.3%)	14 (66.6%)	6.348	0.04*
Irregular cycles	3 (8.1%)	34 (91.9%)		
Postmenopausal women	5 (14.7%)	29 (85.3%)		
Maternal history				
No significant history	12 (27.9%)	31 (72.1%)	7.98	0.04**
Preterm delivery	1 (5.5%)	17 (94.5%)		
Abortions	1 (5.8%)	16 (94.2%)		
Infertility	1 (7.1%)	13 (92.9%)		

## Discussion

In a cross-sectional descriptive study done at Sembakkam village, with a community, the focus was placed on women who were at least 18 years old. A variety of techniques were used to get a complete picture of women's substance use [13,14]. A multistage sampling procedure was used, and nine women participated in the key informant interviews that came after this. To determine the causes of tobacco use, as well as the steps taken to stop it, manual content analysis was carried out besides statistical techniques. The estimated prevalence of tobacco use among women was 15.2%. Women in the lower socioeconomic status group had a higher prevalence of tobacco use compared to those in the middle and high socioeconomic status groups. Thirty-two (100%) women were using SLT products. Moreover, 28 (87.5%) of the women did not want to quit the use of SLT. Similar findings were also found in a study by Bharati et al., which recommends that policy changes concentrating on the workplace are necessary, as the workplace may play a role in the commencement of SLT use [15].

In a community-based cross-sectional study done at Warangal, the prevalence of SLT use was 57%, and the most popular type of tobacco used was tobacco with a pan (40.3%) [16]. Sixty-one percent of homemakers reported exposure to second-hand smoke. Peer pressure was the most frequent justification for the beginning. A high incidence of SLT use was observed in women who were unemployed and illiterate. Similar results were observed in our study, where illiterates and the unemployed had a high dependence. A study by Prabhakar et al. also supports these findings [17].

In a study conducted at the field practicing area of Koppal's Irkalgad Primary Health Centre, a cross-sectional investigation was carried out, which included 829 women using a cluster sampling technique. It was found that 17.85% of SLT products were ingested, with tobacco and betel quid being the most popular combination. A strong correlation was established between the study participants' age, literacy level, marital status, employment position, and tobacco intake [18].

A community-based cross-sectional study was carried out with 1,200 rural women living in Belgaum, Karnataka's Primary Health Centre (PHC), who were between the ages of 15 and 49. A substantial correlation was discovered between tobacco use, dysmenorrhea, and irregular menstrual cycles [19]. Similar findings were observed in our study, where irregular cycles were more common among women.

Using the six-question Fagerstrom Test for Nicotine Dependence for Smokeless Tobacco (FTND-ST), the main goal of Uday et al. was to evaluate nicotine dependence in a group of SLT users. The three groups are Group 1, which consists solely of pan masala and gutka chewers; Group 2, which consists solely of Hans users; and Group 3, which consists solely of betel quid and SLT chewers. Only individuals aged 21-70 who used SLT were chosen at random. There are 100 patients in the entire sample. Compared to Hans and betel quid, chewers of SLT, such as pan masala, were found to have a higher mean nicotine dependence Fagerstrom score [1].

In a study by Sharma et al., tobacco smokers and SLT users had higher odds of being illiterate compared to their counterparts (odds ratio [OR] for smoking tobacco = 1.2; for SLT = 1.5) [20]. Notably, before the age of 17, a higher percentage of illiterate individuals began using SLT (OR = 1.3; 1.21-1.44) and smoking tobacco (OR = 1.1; 1.02-1.26). Individuals without formal education had lower odds of trying to quit smoking and were also less likely to consider quitting in the near future. Similarly, in our study, illiterates have been found to have high dependence.

The systematic review comprising 80 studies provided 121 risk estimates for different cancers [21]. Most of the studies from the Eastern Mediterranean Region (EMR) and South-East Asian Region (SEAR) showed a significant positive association between SLT use and pancreatic cancer (OR: between 1.6 and 2.1), while studies from the European Region (EUR) reported a positive association with oral cancer (OR: between 1.48 and 27.4). Variations were observed in the associations between different cancers and specific SLT products based on their nature, methods of use, and inherent toxicity. Similarly, in our study, oral cancer, oral submucous fibrosis, and pancreatic cancer were more common among tobacco users.

A study by Nargis et al. revealed that adults of lower socioeconomic groups had a lesser prevalence of SLT use, which is due to the increasing prices and taxation of tobacco use [22]. These findings contrast our study in which lower-class women were found to have a high dependence. This may be because of the less awareness among women in the lower socioeconomic classes.

Research by Dwivedi et al. showed that compared to families who do not use tobacco, parents and siblings of coronary artery disease (CAD) patients who use tobacco consume more tobacco [23]. Additionally, children from tobacco-using families are more likely to use tobacco products than those from non-user homes. In contrast, in our study, women of nuclear families are found to have high dependence. Loneliness in nuclear families might be the reason for increased dependence among them.

Limitations

These include potential recall and social desirability bias because of self-reporting of nicotine use and the cross-sectional nature of the design, which limits causal inference. Considering the constraints on resources, we used convenient sampling to conduct this study in selected rural areas of the Kolar district. As many demographic factors contribute to the use of SLT, a study of more variables may contribute to a better understanding of the influencing factors of SLT use among women. Examination of other psychosocial factors contributing to nicotine use, such as stress, social isolation, cultural norms, and economic pressures, can shed light on the other factors influencing nicotine use. Investigation of the longitudinal effects of nicotine use on psychiatric outcomes over time can provide insights into the progression of psychiatric symptoms and addiction severity among rural women using nicotine.

## Conclusions

This study provides valuable insights into the patterns of SLT use; identifies demographic factors influencing dependence such as uneducated women, unemployed women, and women from lower socioeconomic classes; and underscores the significant health hazards such as irregular cycles and infertility associated with this form of tobacco consumption. These results are essential for developing focused interventions meant to lower the use of SLT and enhance public health outcomes. Gaining insight into the contributing elements aids in patient counseling and improves patient outcomes. Intervention strategies that integrate mental health support with tobacco cessation programs should be developed and evaluated. Culturally sensitive approaches tailored to the rural context can enhance engagement and effectiveness of treatments. These interventions can be executed by implementing an awareness campaign to enhance knowledge and by organizing screening camps to identify early signs of dependence.

## Figures and Tables

**Figure 6:**
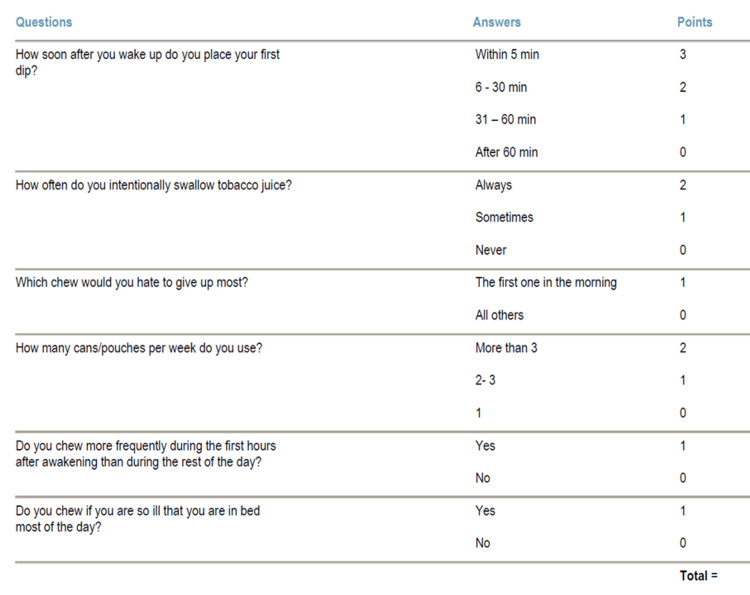
Fagerstrom Nicotine Dependence Scale-Smokeless Tobacco (FTND-ST)

## Appendices

Proforma

1. Name -

2. Age in completed years -

3. Educational qualification - Uneducated/Primary school/High school/Higher Secondary/Graduate

4. Marital status - Married/Unmarried/Widow

5. Socio-economical status - Upper class/Lower class

6. Employed/Unemployed

7. Family - Nuclear/Joint

8. Smokeless tobacco use - Chewing/Snuff/Dip

9. Family history of tobacco use - Yes/No

10. Religion - Hindu/Muslim/Christian/Others

11. History of alcohol consumption - Yes/No

12. Medical illness - Pancreatic cancer/Esophageal cancer/Oral cancer/Oral submucous fibrosis/PCOD/Thyroid disorder/Hypertension/DM/Nil

13. Menstrual history - Regular/Irregular

14. Maternal history - Preterm delivery/Abortions/Infertility/Others/Nil

15. Attempt to quit SLT- Yes/No

16. Reason to quit SLT - Financial reasons, health issues, and pressure from family members

17. Relapse - Yes/No

18. Reason for relapse - Stress/Cravings

Fagerstrom Nicotine Dependence Scale-Smokeless Tobacco

Scoring instructions

Add up responses to all items. A score of 5 or more indicates a significant dependence, while a score of 4 or less shows a low-to-moderate dependence.

## References

[1] Uday HS, Thangavelu RP, Mohan KR, Fenn SM, Appusamy K (2023). Evaluation of nicotine dependence among smokeless tobacco users using the Fagerstrom nicotine dependence scale for smokeless tobacco. Cureus.

[2] Jung-Choi KH, Khang YH, Cho HJ (2012). Hidden female smokers in Asia: a comparison of self-reported with cotinine-verified smoking prevalence rates in representative national data from an Asian population. Tob Control.

[3] Kathirvel S, Thakur JS, Sharma S (2014). Women and tobacco: a cross sectional study from North India. Indian J Cancer.

[4] Schensul JJ, Begum S, Nair S, Oncken C (2018). Challenges in Indian women’s readiness to quit smokeless tobacco use. Asian Pac J Cancer Prev.

[5] Kadidal Kadidal, DHNS A (2024). Tobacco consumption on the rise in rural women: report. Deccan Her.

[6] Jitenkumar singh K, Haobijam N, Nair S (2021). Smokeless tobacco use among women in northeastern states, India: a study of spatial clustering and its determinants using National Family Health Survey-4 data. Clin Epidemiol Glob Health.

[7] Rodríguez-Bolaños R, Caballero M, Ponciano-Rodríguez G, González-Robledo LM, Cartujano-Barrera F, Reynales-Shigematsu LM, Cupertino AP (2021). Gender-related beliefs and attitudes about tobacco use and smoking cessation in Mexico. Health Psychol Behav Med.

[8] Association of Women's Health Obstetric and Neonatal Nurses (2021). Perinatal clinical nurse educator: clinical competencies and education guide. J Obstet Gynecol Neonatal Nurs.

[9] Goyal LD, Verma M, Garg P, Bhatt G (2022). Variations in the patterns of tobacco usage among indian females - findings from the global adult tobacco survey India. BMC Womens Health.

[10] Dasgupta A, Pal M, Paul B, Bandyopadhyay L (2018). Predictors of smokeless tobacco consumption among women: a community based study in a slum of Kolkata. Int J Community Med Public Health.

[11] IARC Working Group on the Evaluation of Carcinogenic Risks to Humans, International Agency for Research on Cancer (2007). Smokeless Tobacco and Some Tobacco-Specific N-Nitrosamines.

[12] Sharma MK, Suman LN, Srivastava K, Suma N, Vishwakarma A (2021). Psychometric properties of Fagerstrom Test of Nicotine Dependence: a systematic review. Ind Psychiatry J.

[13] Joseph I, Rooban T, Ranganathan K (2017). Tobacco use, oral cancer screening, and oral disease burden in Indian women. Indian J Dent Res.

[14] Shrivastava SR, Shrivastava PS (2020). Estimation of the prevalence of tobacco consumption among rural women in South India using mixed methods analysis. Indian J Community Med.

[15] Bharati B, Sahu KS, Pati S (2023). Prevalence of smokeless tobacco use in India and its association with various occupations: a LASI study. Front Public Health.

[16] Lingala M, Simon S, Bhagath Bhagath, Kavitha Kavitha (2021). Prevalence of smokeless tobacco consumption among women of rural Telangana. Int J Community Med Public Health.

[17] Prabhakar B, Narake SS, Pednekar MS (2012). Social disparities in tobacco use in India: the roles of occupation, education and gender. Indian J Cancer.

[18] Yuvaraj BY, Mane VP, Anilkumar L, Biradar M, Nayaka V, Sreenivasamurthy R (2020). Prevalence of consumption of smokeless tobacco products and exposure to second-hand smoke among women in the reproductive age group in a rural area of Koppal, Karnataka. Indian J Community Med.

[19] Kulkarni N, Hawal N, Naik VA (2018). Tobacco use among rural women in reproductive age group and its association with regularity of menstrual cycles and dysmenorrhoea: a community based cross sectional study. Int J Community Med Public Health.

[20] Sharma D, Goel S, Lal P (2017). Education differential in relation to tobacco use and its predictors across different regions of India. Indian J Cancer.

[21] Gupta S, Gupta R, Sinha DN, Mehrotra R (2018). Relationship between type of smokeless tobacco & risk of cancer: a systematic review. Indian J Med Res.

[22] Nargis N, Yong HH, Driezen P (2019). Socioeconomic patterns of smoking cessation behavior in low and middle-income countries: emerging evidence from the Global Adult Tobacco Surveys and International Tobacco Control Surveys. PLoS One.

[23] Dwivedi S, Aggarwal A, Singh N, Aggarwal S, Sharma V (2013). Role of family milieu in tobacco addiction: a study in a tertiary-care institution in India. J Health Popul Nutr.

